# Temporal Dynamic Alterations of Regional Homogeneity in Parkinson’s Disease: A Resting-State fMRI Study

**DOI:** 10.3390/biom13060888

**Published:** 2023-05-25

**Authors:** Kai Li, Yuan Tian, Haibo Chen, Xinxin Ma, Shuhua Li, Chunmei Li, Shaohui Wu, Fengzhi Liu, Yu Du, Wen Su

**Affiliations:** 1 Department of Neurology, National Center of Gerontology, Institute of Geriatric Medicine, Beijing Hospital, Chinese Academy of Medical Sciences, No. 1 Dahua Road, Dong Dan, Beijing 100730, China; 2 Graduate School, Peking Union Medical College, Dongcheng, Beijing 100730, China; 3 Department of Radiology, National Center of Gerontology, Institute of Geriatric Medicine, Beijing Hospital, Chinese Academy of Medical Sciences, No. 1 Dahua Road, Dong Dan, Beijing 100730, China

**Keywords:** Parkinson’s disease, resting-state fMRI, dynamic brain activity, regional homogeneity, support vector machine, neurodegenerative diseases, movement disorders, neuroimaging, depression, anxiety

## Abstract

Brain activity is time varying and dynamic, even in the resting state. However, little attention has been paid to the dynamic alterations in regional brain activity in Parkinson’s disease (PD). We aimed to test for differences in dynamic regional homogeneity (dReHo) between PD patients and healthy controls (HCs) and to further investigate the pathophysiological meaning of this altered dReHo in PD. We included 57 PD patients and 31 HCs with rs-fMRI scans and neuropsychological examinations. Then, ReHo and dReHo were calculated for all subjects. We compared ReHo and dReHo between PD patients and HCs and then analyzed the associations between altered dReHo variability and clinical/neuropsychological measurements. Support vector machines (SVMs) were also used to assist in differentiating PD patients from HCs using the classification values of dReHo. The results showed that PD patients had increased ReHo in the bilateral medial temporal lobe and decreased ReHo in the right posterior cerebellar lobe, right precentral gyrus, and supplementary motor area, compared with controls. The coefficient of variation (CV) of dReHo was considerably higher in the precuneus in PD patients compared with HCs, and the CV of dReHo in the precuneus was found to be highly associated with HAMD, HAMA, and NMSQ scores. Multiple linear regression analysis controlling for demographic, clinical, and neuropsychiatric variables confirmed the association between altered dReHo and HAMD. Using the leave-one-out cross validation procedure, 98% (*p* < 0.001) of individuals were properly identified using the SVM classifier. These results provide new evidence for the aberrant resting-state brain activity in the precuneus of PD patients and its role in neuropsychiatric symptoms in PD.

## 1. Introduction

Parkinson’s disease (PD) is a progressive neurodegenerative disorder that mostly occurs in aging people. It is characterized by several clinical features, including motor dysfunction, such as bradykinesia, postural instability, resting tremor, and rigidity, accompanied by many nonmotor symptoms, which result in serious health and social problems [1,2]. Despite significant research progress over the last few decades, the pathophysiological mechanisms of PD are still not completely understood [3,4].

Advancements in neuroimaging techniques have made it easier to learn more about PD. In particular, the use of resting-state functional magnetic resonance imaging (rs-fMRI) to study spontaneous cerebral activity in PD has grown increasingly in recent years [5]. One measure, known as regional homogeneity (ReHo), uses Kendall’s coefficient of concordance (KCC) to measure similarities between a given voxel and nearby voxels to detect the regional functional integration of spontaneous neuronal activity [6]. ReHo measures the local synchronization of spontaneous neuronal activity and does not directly correspond to an increase or decrease in overall activity but rather provides information on the organization of brain activity [6]. On the basis of this, a ReHo map of the KCC value distribution can be produced. ReHo has been widely used to study aberrant brain functional activity in neurological disorders, including PD, and there have been various reports about studies of abnormal ReHo in PD [7,8,9]. A meta-analysis of ReHo research found that intrinsic brain activity exhibits a pattern of dysfunction and adjustment that mostly involves default mode networks (DMN) and motor networks [10].

Evidence has emerged recently indicating that brain activity is time varying, even in the resting state [11]. Brain activity is dynamic throughout the scan period, but static analysis overlooks these dynamic fluctuations. Dynamic measures such as dynamic ReHo (dReHo) can provide new information on the dynamic nature of neural activity [12,13,14,15,16]. DReHo is a valuable measure for investigating abnormal brain activity in patients with attention-deficit hyperactivity disorder [12], stroke [13], amyotrophic lateral sclerosis [14], trigeminal neuralgia [15], and mild cognitive impairment [16]. In PD patients’ dynamic regional brain activity assessments, dALFF changes in the left precuneus have already been reported [16]. However, there has been no research about dReHo analysis in PD. Therefore, dReHo was used as a dynamic characteristic of spontaneous functional brain activity in both PD and healthy controls (HCs) in the current study. We wanted to see if patients with PD had altered dReHo levels and, if so, whether the variation corresponded to changes in clinical measurements. Additionally, we also compared ReHo between the two groups to observe the different results provided by comparing ReHo and dReHo between the two groups using two distinct analytic methods.

Although the primary objective of this study was to investigate dynamic abnormalities in brain function in Parkinson’s disease and the pathophysiological significance behind these abnormalities, we also attempted to utilize these dynamic brain function abnormalities for diagnostic analysis. This approach aimed to provide new insights for future diagnostic research in the field. It is possible to classify and predict individuals with high accuracy using support vector machines (SVMs). Combined with measurements of brain dynamic functional connectivity (dFC), they have been used to classify Alzheimer’s disease (AD) and mild cognitive impairment (MCI) [17,18]. Furthermore, in a previous study, SVMs were used to differentiate PD patients from HCs using the dALFF variation classification [16]. In the present study, we also implemented SVMs to investigate whether dReHo could differentiate PD patients from HCs at an individual level.

## 2. Materials and Methods

### 2.1. Participants

The present study recruited 67 patients who were matched for age, gender, and general cognitive status with 34 HCs; none of the HCs had a history of neurological or psychiatric disorders before being enrolled in the study. Table 1 shows a summary of the demographic and clinical data of the included participants. All patients were diagnosed using the Parkinson’s Disease Society of the United Kingdom brain bank diagnostic criteria [19]. We ruled out dementia [20], moderate to severe head tremors, other neurological and psychiatric disorders, including alcohol and drug abuse, and deep brain stimulation. People who were left-handed were also excluded. All PD patients underwent an MRI scan, as well as motor and nonmotor function assessments, in a practically defined “off” state after withdrawing all antiparkinsonian medications for 12 h. The researchers gathered demographic and medical data from all participants. Hoehn-Yahr (H-Y) staging, the Unified Parkinson’s Disease Rating Scale (UPDRS), the Mini-Mental State Examination (MMSE), the Non-Motor Symptoms Questionnaire (NMSQ), the Hamilton Anxiety Rating Scale (HAMA), and the Hamilton Depression Rating Scale (HAMD) were all employed to evaluate PD patients. The control group was also assessed with the MMSE. The Declaration of Helsinki was adhered to throughout the course of this investigation. All participants agreed to take part in the research in writing before their enrollment. The ethics committee of Beijing Hospital approved the research.

### 2.2. MRI Data Acquisition

A 3.0-T MRI scanner (Achieva TX; Philips Medical Systems, Best, The Netherlands) was used at Beijing Hospital to perform all MRI scans. We used foam padding and headphones to restrict head movement and reduce the noise of scanning. During the scan, the participants were instructed to lie still, relax, keep their eyes closed, remain awake, and not focus on any specific thoughts. T1-weighted images with high resolution (three-dimensional turbo field echo) were created using the following settings: echo time (TE) = 3.0 ms, repetition time (TR) = 7.4 ms, flip angle = 8°, field of view (FOV) = 240 × 240 mm, voxel dimensions = 0.94 × 0.94 × 1.20 mm, matrix size = 256 × 256, slice thickness = 1.2 mm, slices = 140. The following parameters were used to create functional images: TE = 35 ms, TR = 3000 ms, matrix size = 64 × 64, flip angle = 90°, FOV = 240 × 240 mm, voxel dimensions = 3.75 × 3.75 × 4.00 mm, slice thickness = 4 mm, slices = 33, time points = 210.

### 2.3. Rs-fMRI Data Preprocessing

The functional images were preprocessed in MATLAB (MathWorks, Inc., Natick, MA, USA) using RESTplus V 1.2 [21] and the SPM12 package (www.fifil.ion.ucl.ac.uk/spm (accessed on 27 August 2018)). For signal acclimatization and equilibrium of the participants, the preprocessing pipeline excluded the first 10 volumes. The remaining 200 time points had their slice timing adjusted. We paid attention to head motion when realigning, ruling out motion greater than 2 mm displacement or 2° rotation. Functional images were normalized to the Montreal Neurological Institute (MNI) template (using DARTEL) and co-registered with structural T1 images [22]. They were then resliced to a 3 × 3 × 3 mm^3^ resolution. Detrending was used to lessen systematic shift. The Friston-24 head motion parameters [23], cerebrospinal fluid, and white matter signals were controlled using nuisance covariates regression.

### 2.4. ReHo Calculation

ReHo maps were generated using RESTPlus V 1.2, following a previously published procedure [6]. Kendall’s coefficient of concordance (KCC) was computed for the time series of each voxel and its 26 nearest neighboring voxels in a voxel-wise manner across the entire brain. To standardize the results, the KCC of each voxel was divided by the average KCC of the entire brain, resulting in normalized ReHo maps. Finally, the ReHo maps were smoothed using a Gaussian kernel (6 mm full-width-half-maximum, FWHM).

### 2.5. DReHo Calculation

Using temporal dynamic analysis (TDA) toolkits based on DPABI V4.3 [24], dynamic regional metric data were analyzed, which is a data-driven approach [25]. Within a predetermined temporal window, ReHo was calculated according to the sliding window analysis’s specifications. Over the course of the entire brain, Kendall’s coefficient of concordance (KCC) was calculated between the time series of each voxel and the time series of its nearest 26 neighbor voxels [6]. A window length that is either short or too lengthy may not be able to detect dynamic activity or allow for a robust assessment of dynamic changes. Previous research established the optimal window length range as 10–75 TR, step = 1 TR [11,26]. In the present study, dynamic analysis was conducted using a 30 TR sliding window with a shifting step size of 1 TR. One hundred and seventy-one windows were created from 200 time intervals. In addition, in order to exclude the effect of window width on the results, the window width was set to 25/35 TR to repeat all the calculations in the paper.

Within each time window, ReHo was calculated. Then, in order to analyze the variability of ReHo, we produced coefficient of variation (CV) maps of ReHo for each individual. DReHo was calculated by computing the CV of the ReHo at each voxel using 171 time windows, yielding dReHo graphs for each subject. Finally, the individual dReHo maps were spatially smoothed (full-width-half-maximum (FWHM) of the Gaussian kernel = 6 mm).

### 2.6. Support Vector Machine Analysis

We applied SVMs to classify data using intergroup differences in the CV maps of dReHo in the area that showed significant differences between the two groups. The K-nearest neighbor (KNN) imputation with N = 3 was employed to apply the feature imputation to complete the missing values. This feature was used to establish the available models with the SVMs. The sample contained 88 participants and was divided into a training group of 71 participants and a testing group of 17 participants. The participants in the two groups had comparable ages, gender ratios, disease durations, and Hoehn-Yahr stages. To obtain an idea of how general our classifier could be, we ran it through the leave-one-out cross validation (LOOCV) method. An analysis of the permutation test showed that this classification accuracy was statistically significant [27]. Every 5000 times the classifier randomly assigned PD and HC labels to the training subjects, the permutation test was repeated, and the classification process was repeated. The classifier’s overall sensitivity, specificity, accuracy, and ROC were then calculated to test its performance.

### 2.7. Statistical Analysis

Clinical data were analyzed using SPSS (Version 23.0. Armonk, NY, USA: IBM Corp). The Kolmogorov–Smirnov test was used to determine data normality. The intergroup differences in age and MMSE scores were investigated using two-sample *t*-tests or the Mann–Whitney U test. In terms of gender differences, the chi-square test was employed.

DPABI V4.3 was used to compare ReHo and dReHo between the PD and HC groups. Differences in ReHo and dReHo were compared using two-sample *t*-test analysis, in which age and gray matter density (GMD) were included as covariates. Multiple comparisons were corrected using the Gaussian random field (GRF) method (voxel level, *p* < 0.001; cluster level, *p* < 0.05). Spearman correlation analysis was employed to calculate the associations between altered dReHo variability and H-Y staging, UPDRS, disease duration, and MMSE/HAMD/HAMA/NMSQ scores. To control for the effect of other parameters on the significant correlations, we further performed partial correlation and multiple linear regression analyses.

R version 3.6.0 was used for support vector machine analysis. A *p*-value of <0.05 was considered statistically significant if not specified.

## 3. Results

### 3.1. Clinical and Demographic Characteristics

Finally, the analyses involved 57 PD patients and 31 HCs. Four patients with PD and two HCs were excluded because they were left-handed. As a result of excessive head motion, five people with PD and one HC were excluded. Poor image quality led to the exclusion of one PD patient.

Table 1 shows a summary of the demographic and clinical data. In terms of age, gender, and MMSE scores, the results did not show any significant differences between the PD patients and HCs (*p* > 0.05).

### 3.2. Difference in ReHo between PD Patients and Controls

**Figure 1:**
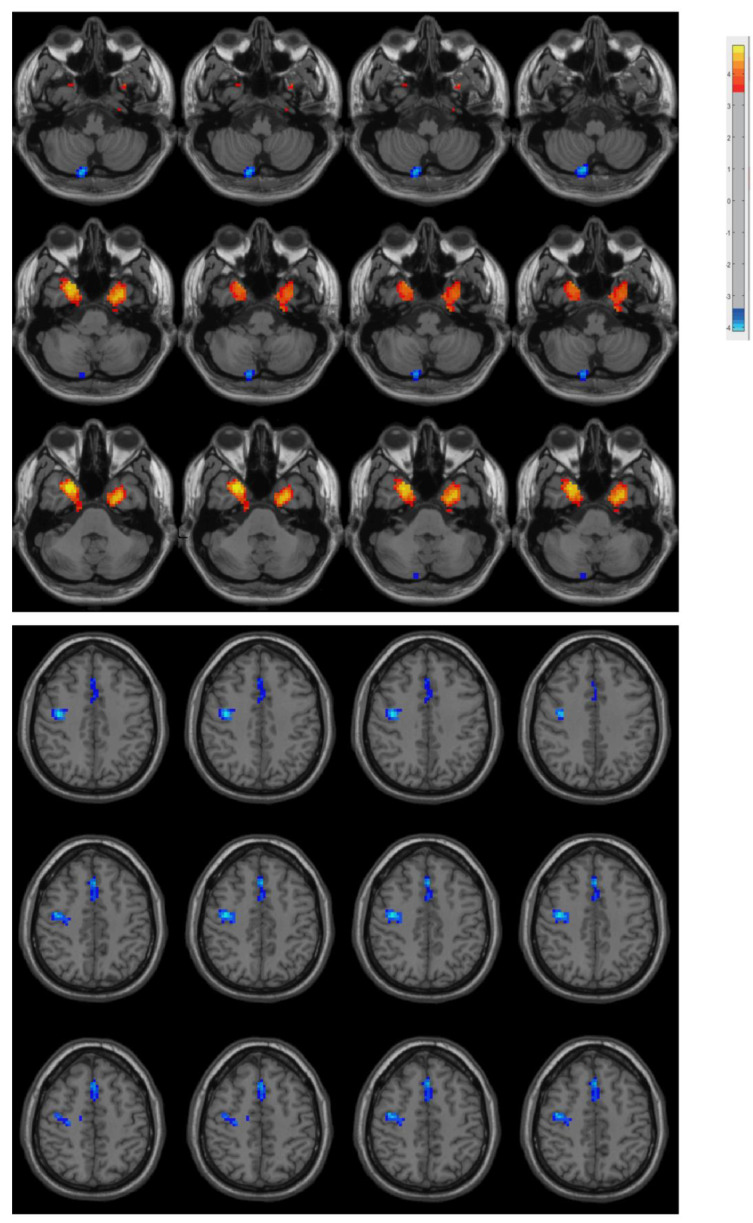
Differences between groups in terms of ReHo. Compared with the controls, the PD patients had increased ReHo in the bilateral medial temporal lobe and decreased ReHo in the right posterior cerebellar lobe, right precentral gyrus, and supplementary motor area.

The PD patients had increased ReHo in the bilateral medial temporal lobe and decreased ReHo in the right posterior cerebellar lobe, right precentral gyrus, and supplementary motor area compared with the controls (Figure 1 and Table 2).

### 3.3. DReHo Analysis and Correlation Analysis

**Figure 2:**
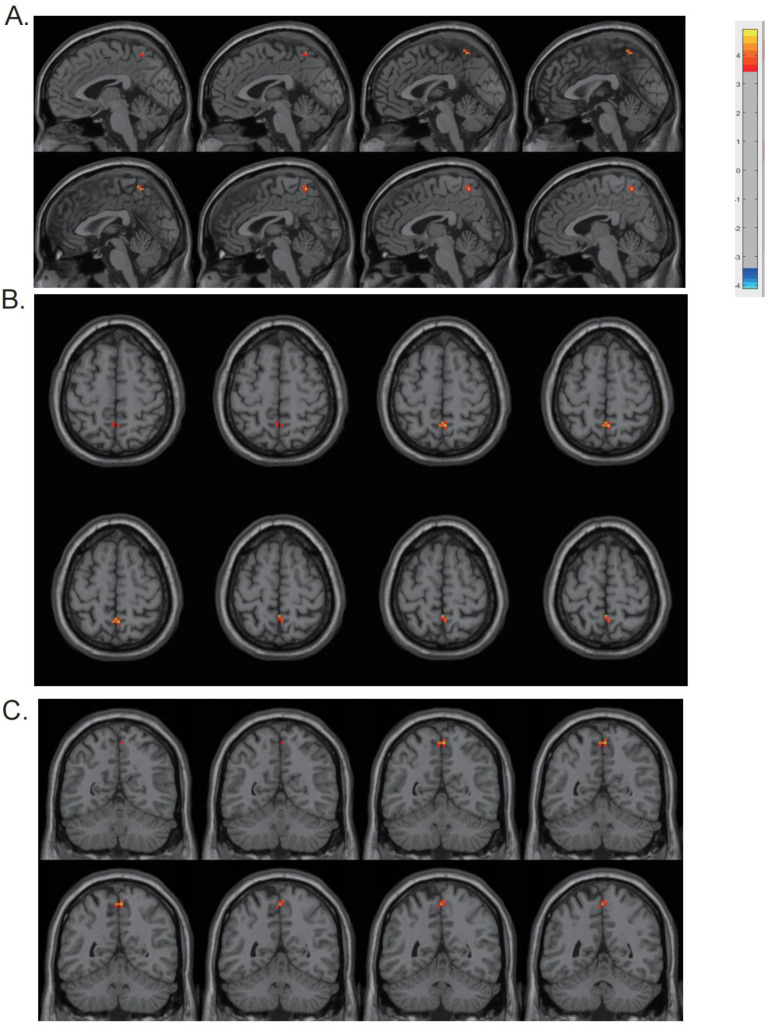
Differences between groups in terms of dReHo variability. The CV of dReHo in the precuneus was found to be higher in the PD patients, as shown in sagittal (**A**), transverse (**B**), and coronal (**C**) views.

**Figure 3:**
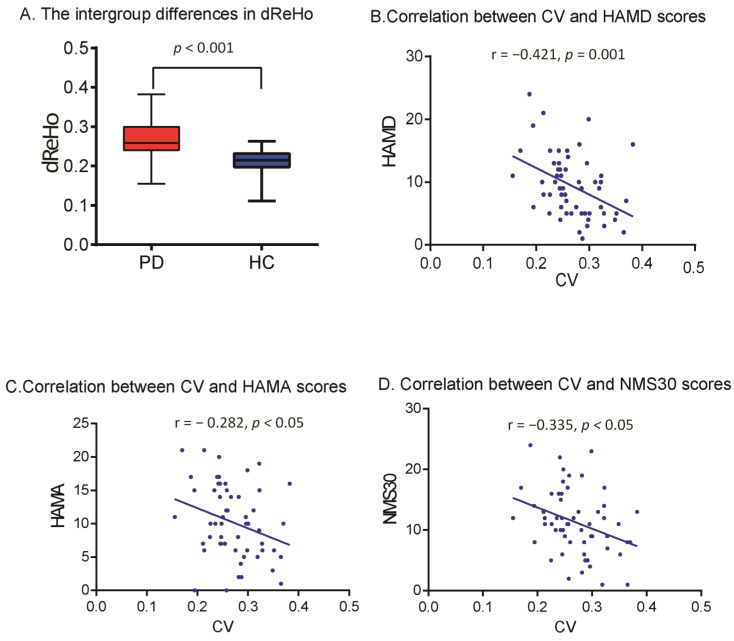
To compare the CV values of dReHo in the precuneus of the two groups (**A**), we used box plots with whiskers (min–max) and applied scatterplots to examine how the precuneus’s CV value related to HAMD (**B**), HAMA (**C**), and NMSQ scores (**D**).

The CV of dReHo was substantially higher in the precuneus in PD patients than in HCs (*p* < 0.001) (Table 3 and Figure 2). The cluster size was 13 voxels. The CV of dReHo in the precuneus was significantly correlated with HAMD, HAMA, and NMSQ scores (*r* = −0.441, −0.312, and −0.345; *p* = 0.001, 0.018, and 0.009) in PD patients (Figure 3). There were no significant correlations between altered dReHo and disease duration, disease severity (H-Y staging and UPDRS score), or MMSE score. To verify whether the correlations between altered dReHo and HAMA, HAMD, and NMSQ scores were due to a common association between disease progression, we performed partial correlation analyses while controlling for disease duration as a covariate, and the results remained consistent with those obtained without controlling for disease duration. In addition, for better control of the contribution of other parameters, we performed multiple linear regression analysis, with altered dReHo as the dependent variable and age, education, head motion (mean framewise displacement (FD), introduced by Jenkinson et al. [28]), and UPDRS, MMSE, HAMA, HAMD, and NMS scores as independent variables. In the multiple linear regression analysis, we found that the HAMD score was the only parameter with statistical significance. The standardized coefficients beta value was −0.403, the significance (sig.) was 0.04, and the 95% confidence interval ranged from −0.008 to 0. This result suggested that there was a significant association between altered dReHo and HAMD score after controlling for other factors, such as UPDRS, head motion parameters, age, and education level.

The results using the window lengths of 25/35 TR were almost the same as those of 30 TR, which further confirmed the reliability of our results. The results using the window lengths of 25/35 TR are detailed in the Supplementary Materials.

### 3.4. Classification Results

**Figure 4:**
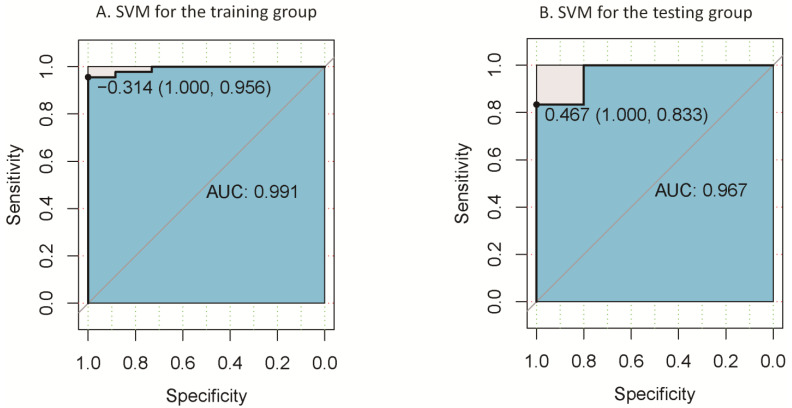
Leave-one-out cross validation (**A**) was employed in the PD group, as well as nested 10-fold validation; (**B**) yielded classification accuracy of altered dynamic ReHo in the precuneus.

The above results showed that the region with between-group differences was located in the precuneus. We then saved this cluster as a mask. Following this, we used DPABI to extract the signal data of this area in each participant. Finally, we used SVMs to investigate whether dReHo could differentiate PD patients from HCs. Figure 4 depicts the outcomes. The linear SVM classifier’s accuracy trained with LOOCV was 98%, with 100% sensitivity and 95.6% specificity (*p* < 0.001, non-parametric permutation approach). The area under the classifier’s receiver operating characteristic (ROC) curve was 0.991. We used the validation group to test the classification method’s reliability, and the area under the classifier’s ROC curve was 0.967.

## 4. Discussion

In this study, we mainly explored the dynamic neural activity pattern of PD with the new TDA method of dReHo. The comparison of ReHo and dReHo between the PD patients and controls obtained different results. The CV of dReHo was obviously increased in the precuneus of PD patients, which was associated with depressive symptoms.

The present study found increased ReHo in the bilateral medial temporal lobe and decreased ReHo in the right posterior cerebellar lobe, right precentral gyrus, and medial frontal gyrus in PD patients compared with controls. These findings are consistent with previous reports [7,8,29,30,31,32,33,34]. The cerebellum, precentral gyrus, and supplementary motor area are closely associated with motor function, and the motor system is impaired in PD. Previous research has reported that ReHo is reduced in these areas among PD patients [7,8,29,31,32,33,34,35]. The medial temporal lobe is involved in Parkinson’s disease at Braak stage 4, closely following substantia nigra involvement, which leads to motor symptoms in Braak stage 3 [36]. Altered ReHo in this area has also been commonly reported to be abnormal in PD patients and is associated with neuropsychiatric symptoms, especially depression [31,34,35].

In the present study, analysis of dReHo obtained different results from ReHo. Because ReHo represents the local synchronization of spontaneous neuronal activity of an entire period, while dReHo reflects fluctuations in ReHo by dividing the period into many short time windows, they have different physiological mechanisms. DReHo may detect altered brain activity from another perspective [12,13,14,15,16]. In recent years, dReHo has gradually been used in the study of neurodegenerative diseases. In a study on mild cognitive impairment (MCI) (with depression or without depression), dynamical measurements offered improved insight into the association between memory deficits and depressive symptoms [16]. The latest findings demonstrated that dReHo may be a useful biomarker for the early detection and diagnosis of diseases such as amyotrophic lateral sclerosis (ALS) [14]. Furthermore, a previous study showed that dALFF in the left precuneus of PD patients differed significantly from that of HCs, and the CV of dALFF was correlated with the course of the disease [16]. Our new research demonstrated that dReHo was obviously increased in the precuneus of PD patients, which provided meaningful and robust information from a dynamic perspective.

Rich evidence suggests that the precuneus is a critical cerebral region in PD patients. The precuneus is in the posteromedial cortex of the parietal lobe and has been a hot topic of the highest metabolism in cerebral regions, which plays a critical role in motor and cognitive tasks [37]. Additionally, it has been found to have the most obvious decreased metabolism in the posterior cortical region of PD patients when using other neuroimaging technologies [38,39,40]. A number of studies have revealed that the precuneus is closely associated with both motor and nonmotor symptoms in PD [38,40,41,42,43,44,45,46]. It is believed that the precuneus is involved in the default mode network (DMN), and the inter-network connectivity from DMN to motor systems is impaired in PD [41]. Voxel-based morphometry discovered morphological changes in the precuneus of PD patients who had cognitive impairment and isolated apathy; attention and working memory dysfunction were observed in patients with PD who had apathy [43]. Using static brain activity analysis, Hu discovered an association between HAMD score and altered functional connectivity between the precuneus and the left median cingulated cortex (MCC) [44]. Even in the early stages of PD, precuneus cortical thickening has been shown in patients with mild–moderate depression. This finding may point to an early role for this region in the onset of depression in PD patients [45]. Our current research has also discovered significant negative correlations between dReHo and nonmotor symptoms, particularly depression and anxiety. This suggests that changes in dynamical homogeneity in this region may serve as a compensatory mechanism for anxiety and depression symptoms. The precuneus is a perceptual processing center that is linked to visuospatial function, situational memory extraction, and self-awareness [47]. Studies have shown that patients with serious depression have unusually high bilateral precuneus functional connectivity. Antidepressant treatment reduces clinical symptoms while normalizing bilateral precuneus functional connectivity [48]. In conclusion, precuneus functional status is likely to be an imaging tool for measuring the severity of depressive symptoms in PD patients.

Many studies have combined machine learning algorithms with brain DFC networks to classify patients in the early stages of AD. A prior study combined DFC analysis with SVM to classify early MCI patients and healthy controls, with almost 80% accuracy compared to 62–72% accuracy using sFC [17]. Another recent study tested that the SVM classifier correctly classified 80.36% of subjects using the LOOCV method. When using dALFF variation in the left precuneus, it may be possible to distinguish between PD patients and HCs on an individual level [16]. In our investigation, we also attempted to apply SVM analysis. When the LOOCV method was used, the accuracy of this classification was 98%; this result provides insight for future longitudinal cohort studies that could enroll patients with early Parkinson’s disease to identify its diagnostic value.

There are several limitations to our research. First, all of the patients were on long-term medication. Despite the fact that patients underwent fMRI scans 12 h after medication withdrawal, it was not possible to remove the possibility of long-term consequences of therapy. Second, because of the insufficient number of samples, we employed SVM to double-check the classification accuracy. Although the sample size ratio of the PD patients to HCs was close to 2:1, such a sample distribution may have had an impact on the machine learning classification effect. Our ROC curve performed well when we actually trained the model. In future investigations, in order to ensure classification accuracy, a larger sample size and more matched sample size ratio should be used, and a separate test sample should be employed.

## 5. Conclusions

In conclusion, this study sheds light on the dynamic alterations in regional brain activity in PD. Our findings revealed a significant increase in the CV of dReHo in the precuneus of PD patients compared to HCs. Furthermore, the CV of dReHo in the precuneus was found to be strongly associated with HAMD, HAMA, and NMSQ scores, especially depressive scores, highlighting the role of the precuneus in neuropsychiatric symptoms in PD. This study not only provides new evidence for aberrant resting-state brain activity in the precuneus of PD patients from a dynamic perspective but also suggests the potential of dReHo as a valuable imaging tool for understanding the pathophysiological mechanisms of PD and assisting in its diagnosis.

## Figures and Tables

**Table 1:** Clinical and Demographic features of Parkinson’s disease (PD) patients and healthy controls (HCs).

	PD	HCs	*p*-Value
No. of subjects	57	31	
Age	64.16 ± 8.13	62.42 ± 7.19	0.122
Gender (M/F)	28/29	16/15	0.823
Disease duration	6.49 ± 3.59	N/A	N/A
H-Y staging	2.20 ± 0.69	N/A	N/A
UPDRS	49.90 ± 18.82	N/A	N/A
MMSE	28.05 ± 1.95	27.71 ± 2.25	0.528
HAMD	9.30 ± 5.14	N/A	N/A
HAMA	10.21 ± 5.49	N/A	N/A
NMSQ	11.30 ± 5.32	N/A	N/A

PD, Parkinson’s disease; HCs, Healthy controls; M, male; F, female; H-Y staging, Hoehn-Yahr staging; UPDRS, Unified Parkinson’s Disease Rating Scale; MMSE, Mini-Mental Status Examination; HAMA, Hamilton Anxiety Rating Scale; HAMD, Hamilton Depression Rating Scale; NMSQ, Non-Motor Symptoms Questionnaire. Data are presented as range and mean ± SD.

**Table 2:** ReHo differences between PD and HCs.

Region	Cluster Size (Voxel)	MNI (x, y, z)	*t*-Value
Right Posterior Cerebellar Lobe	17	(15, −81, −54)	−5.5695
Left Medial Temporal Lobe	52	(−27, 0, −42)	5.1186
Right Medial Temporal Lobe	74	(27, 9, −39)	5.4905
Right Precentral Gyrus	123	(39, −9, 42)	−5.7469
Supplementary Motor Area	70	(0, 24, 48)	−5.2007

**Table 3:** DReHo differences between PD patients and HCs.

Region	Cluster Size (Voxel)	MNI (x, y, z)	*t*-Value
Precuneus	13	(3, −54, 60)	4.5626

## Data Availability

The data that support the findings of this study are available on request from the corresponding author. The data are not publicly available due to their containing information that could compromise the privacy of research participants.

## Supplementary Materials

The following supporting information can be downloaded at https://www.mdpi.com/article/10.3390/biom13060888/s1: Figure S1: dReHo variability differences between the PD and HC groups (Window length = 25TR); Table S1: dReHo differences between PD and HCs; Figure S2: dReHo variability differences between the PD and HC groups (Window length = 35TR); Table S2: dReHo differences between PD and HCs (Window length = 35TR).
