# Supplementary material for: Temporal Dynamic Alterations of Regional Homogeneity in Parkinson’s Disease: A Resting-State fMRI Study
Source: Biomolecules. 2023 May 25;13(6):888. doi: 10.3390/biom13060888 (PMC10296430; doi:10.3390/biom13060888)
Supplement: Supplementary file 1 [file biomolecules-13-00888-s001.zip › biomolecules-2225610-supplementary.pdf]

Supplementary Material

Choice of window length

In fact, too short a window length may not allow robust estimation of dynamic changes, and too long a window length may not be able to detect dynamic activity. Previous studies used the range of the appropriate window length as 10–75 TR, step = 1 TR (1, 2). Both 2s and 3s are commonly used TR parameters. To maximize the statistical power, a moderate sliding window length of 30 TR (step = 1 TR) was selected.

In addition, we have tried different types of windows, such as 25/35TR, the following are the results of our data analysis with different window widths.

In the window length as 25TR, there are almost the same results which show the increased CV of dReHo in precuneus.

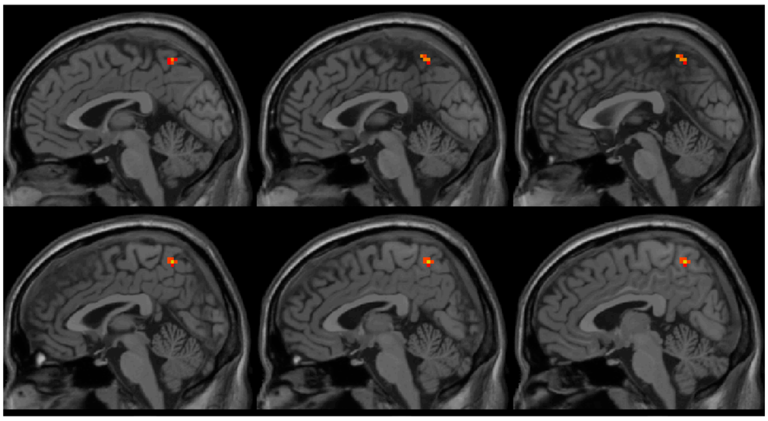

Figure S1. dReHo variability differences between the PD and HC groups (Window length=25TR).

Table S1. dReHo differences between PD and HCs (Window length=25TR)

**Region**	**Cluster size (voxel)**	**MNI (x, y, z)**	***t*-value**
Precuneus	24	(3, -54, 60)	4.7559

In the window length as 35TR, there are the approximately the same results which shows the increaed CV of dReHo in precuneus.

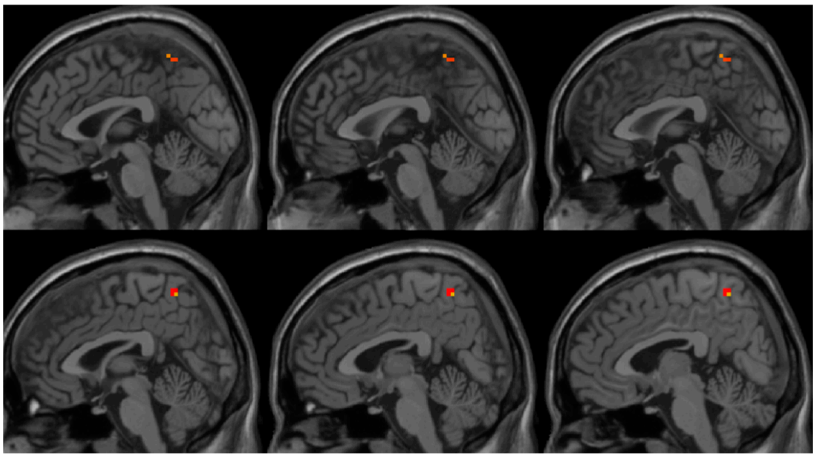

Figure S2. dReHo variability differences between the PD and HC groups (Window length=35TR).

Table S2. dReHo differences between PD and HCs (Window length=35TR**)**

Region	Cluster size (voxel)	MNI (x, y, z)	*t*-value
Precuneus	8	(3, -54, 60)	4.3767

References

1. Liu F, Wang Y, Li M, Wang W, Li R, Zhang Z, et al. Dynamic functional network connectivity in idiopathic generalized epilepsy with generalized tonic-colonic seizure. *Hum Brain Mapp*. (2017) 38:957–73. doi:10.1002/hbm.23430

2. Zalesky A, Breakspear M. Towards a statistical test for functional connectivity dynamics. *Neuroimage*. (2015) 114:466–70. doi: 10.1016/j.neuroimage.2015.03.047
